# Correction: Combined Genetic and High-Throughput Strategies for Molecular Diagnosis of Inherited Retinal Dystrophies

**DOI:** 10.1371/journal.pone.0101641

**Published:** 2014-06-24

**Authors:** The PLOS ONE Staff

## Notice of Republication

This article was republished on May 30, 2014, to replace an incorrectly published version where Table 4 was missing in the XML and PDF of the article. Please download this article again to view the correct version. The originally published, uncorrected article and the republished, corrected article are provided here for reference.

## Supporting Information

File S1 Originally published, uncorrected article.
(PDF)

File S2 Republished corrected article.
(PDF)
