# Supplementary figures and images for: Combined Genetic and High-Throughput Strategies for Molecular Diagnosis of Inherited Retinal Dystrophies
Source: PLoS One. 2014 Feb 7;9(2):e88410. doi: 10.1371/journal.pone.0088410 (PMC3917917; doi:10.1371/journal.pone.0088410)

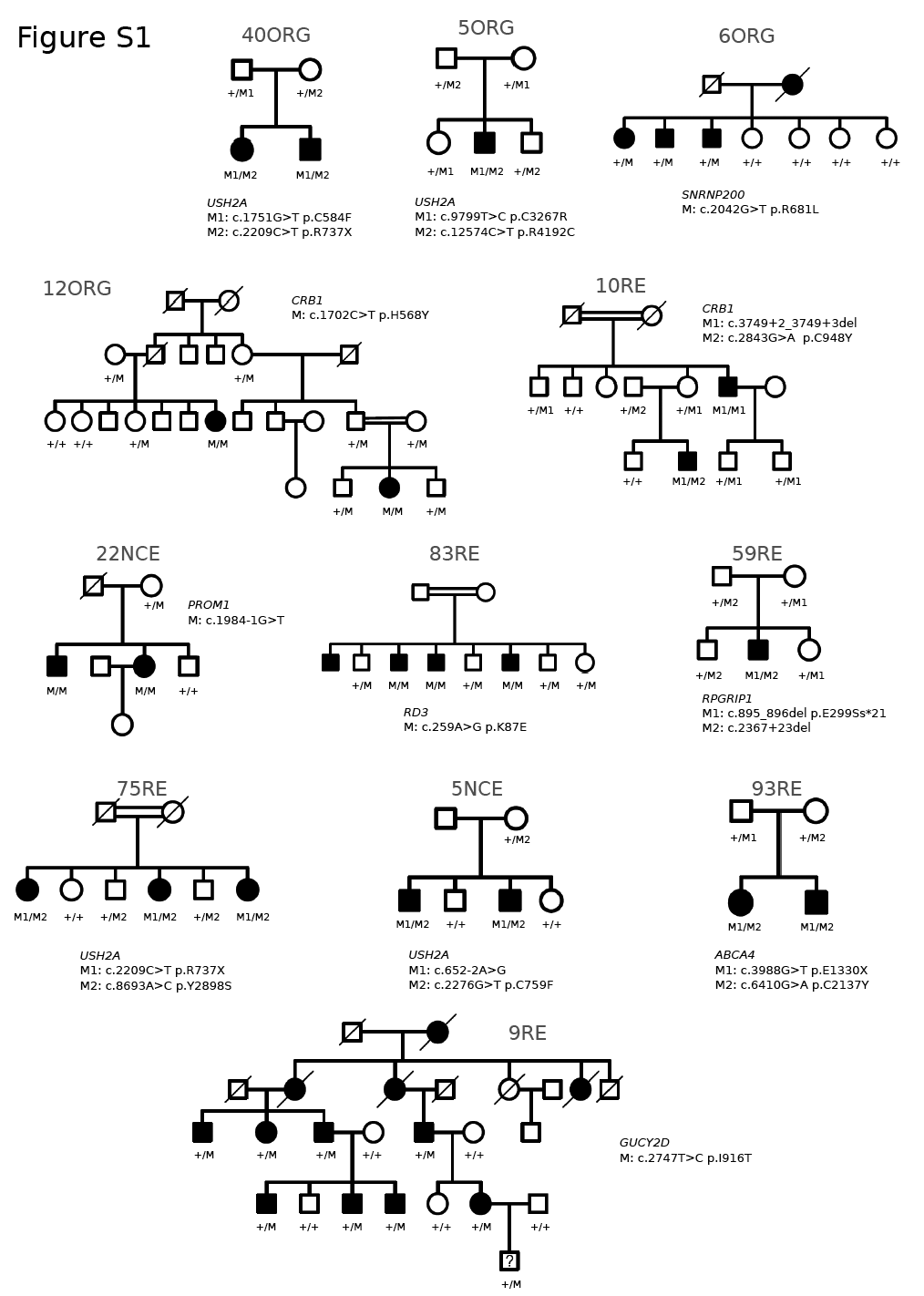

Supplement: Figure S1 — Cosegregation analysis of the novel mutations identified. M: mutation (TIF) [file pone.0088410.s001.tif]
