# Supplementary material for: Correction: Combined Genetic and High-Throughput Strategies for Molecular Diagnosis of Inherited Retinal Dystrophies
Source: PLoS One. 2014 Jun 24;9(6):e101641. doi: 10.1371/journal.pone.0101641 (PMC4069171; doi:10.1371/journal.pone.0101641)
Supplement: File S2 — Republished corrected article. (PDF) [file pone.0101641.s002.pdf]

OPEN ACCESS Freely available online

PLOS ONE

Combined Genetic and High-Throughput Strategies for Molecular Diagnosis of Inherited Retinal Dystrophies

Marta de Castro-Miró[1,2,3], Esther Pomares[1,2¤], Laura Lorés-Motta[1], Raul Tonda[4], Joaquín Dopazo[5,6], Gemma Marfany[1,2,3], Roser Gonzàlez-Duarte[1,2,3]*

1 Departament de Genètica, Facultat de Biologia, Universitat de Barcelona, Barcelona, Spain, **2** Centro de Investigación Biomédica en Red de Enfermedades Raras (CIBERER), Instituto de Salud Carlos III, Barcelona, Spain, **3** Institut de Biomedicina (IBUB), Universitat de Barcelona, Barcelona, Spain, **4** Centre Nacional d'Anàlisi Genòmica, PCB, Barcelona, Spain, **5** Department of Computational Genomics, Centro de Investigación Príncipe Felipe, Valencia, Spain, **6** BIER, Centro de Investigación Biomédica en Red de Enfermedades Raras (CIBERER), Valencia, Spain

Abstract

Most diagnostic laboratories are confronted with the increasing demand for molecular diagnosis from patients and families and the ever-increasing genetic heterogeneity of visual disorders. Concerning Retinal Dystrophies (RD), almost 200 causative genes have been reported to date, and most families carry private mutations. We aimed to approach RD genetic diagnosis using all the available genetic information to prioritize candidates for mutational screening, and then restrict the number of cases to be analyzed by massive sequencing. We constructed and optimized a comprehensive cosegregation RD-chip based on SNP genotyping and haplotype analysis. The RD-chip allows to genotype 768 selected SNPs (closely linked to 100 RD causative genes) in a single cost-, time-effective step. Full diagnosis was attained in 17/36 Spanish pedigrees, yielding 12 new and 12 previously reported mutations in 9 RD genes. The most frequently mutated genes were *USH2A* and *CRB1*. Notably, *RD3*–up to now only associated to Leber Congenital Amaurosis– was identified as causative of Retinitis Pigmentosa. The main assets of the RD-chip are: i) the robustness of the genetic information that underscores the most probable candidates, ii) the invaluable clues in cases of shared haplotypes, which are indicative of a common founder effect, and iii) the detection of extended haplotypes over closely mapping genes, which substantiates cosegregation, although the assumptions in which the genetic analysis is based could exceptionally lead astray. The combination of the genetic approach with whole exome sequencing (WES) greatly increases the diagnosis efficiency, and revealed novel mutations in *USH2A* and *GUCY2D*. Overall, the RD-chip diagnosis efficiency ranges from 16% in dominant, to 80% in consanguineous recessive pedigrees, with an average of 47%, well within the upper range of massive sequencing approaches, highlighting the validity of this time- and cost-effective approach whilst high-throughput methodologies become amenable for routine diagnosis in medium sized labs.

Citation: de Castro-Miró M, Pomares E, Lorés-Motta L, Tonda R, Dopazo J, et al. (2014) Combined Genetic and High-Throughput Strategies for Molecular Diagnosis of Inherited Retinal Dystrophies. PLoS ONE 9(2): e88410. doi:10.1371/journal.pone.0088410

Editor: Hector Escriva, Laboratoire Arago, France

Received November 28, 2013; **Accepted** January 6, 2014; **Published** February 7, 2014

Copyright: © 2014 de Castro-Miró et al. This is an open-access article distributed under the terms of the Creative Commons Attribution License, which permits unrestricted use, distribution, and reproduction in any medium, provided the original author and source are credited.

Funding: MdCM was a recipient of a FPI fellowship from the Spanish MICINN. This study was supported by grants SAF2009-08079 (RGD), BFU2010-15656 (GM), and BIO2011-27069 (JD) (Ministerio de Ciencia e Innovación), SGR2009-1427 (RGD)(Generalitat de Catalunya), CIBERER (U718 and U715), Retina Asturias (RGD) and ONCE (RGD). The funders had no role in study design, data collection and analysis, decision to publish, or preparation of the manuscript.

Competing Interests: No competing interests to be declared.

* E-mail: rgonzalez@ub.edu

¤ Current address: Instituto de Microcirugía Ocular, Barcelona, Spain

Introduction

Retinal dystrophies (RD) are a group of more than 25 genetic visual disorders [1]. Although RDs rank among mendelian rare diseases, taken together, they occur at an estimated prevalence of 1–2 patients per 1000 individuals. In fact, the most frequent form of RD, retinitis pigmentosa (RP), affects 1.5 million individuals worldwide [2]. The clinical traits underlying these disorders disturb from the macular region (central vision) to the outlying retinal area (peripheral vision). In addition, at least 30 different syndromes (such as Usher and Bardet-Biedl) share some of these phenotypic alterations [3,4]. On the genetic side, more than 5000 mutations in almost 200 genes are causative of retinal dystrophies so far [1,5,6]. Yet, around 35% of the cases remain unassigned [7]. The extreme heterogeneity of RDs at the clinical and genetic levels hinders the accurate clinical assessment, patient management, and genetic counseling. Within this context, molecular diagnosis, however challenging, is instrumental to improve the diagnosis and prognosis of RDs and guide future therapies [7–9].

Currently, the most demanding issue in RD molecular diagnosis is the prioritization of methodological strategies, where the main parameters to be balanced are cost, time and yield. These parameters strongly depend on phenotypic clinical assessment, pedigree information, sample availability and methodological resources. Most genetic laboratories resort to direct mutational screening when the clinical traits and/or the genetic information associated to the disease limit the number of candidates to be analyzed. Unfortunately, this is not a common case for RDs, and this type of analysis would imply screening more than 1500 exons. The search for an alternative cost-effective approach is mainly being performed using high-throughput platforms, in particular massive sequencing, which require powerful and sophisticated bioinformatics tools for analyzing and filtering the data [10,11].

To improve diagnosis, we have focused on a comprehensive strategy based on the clinical phenotype and all available genetic data prior to either analyze a reduced manageable number of candidate genes or resort to massive sequencing. We have generated and optimized a SNP-based chip for haplotype cosegregation analysis [12,13] to genotype 7–10 SNP markers of one hundred genes associated to the most prevalent RDs: Cone Dystrophies (CD), Cone-Rod Dystrophies (CRD), Congenital Stationary Night Blindness (CSNB), Leber Congenital Amaurosis (LCA), Macular Degeneration (MD) and RP. Based on this methodology, a multi-tiered approach has been devised to cost-effectively diagnose [14] a panel of 36 Spanish families with non-syndromic retinal dystrophies plus 5 patients with Usher's syndrome. As a result, we have identified the pathogenic mutation of 17 out of the 36 families, and 3 of the 5 isolated Usher patients, overall reporting 14 novel mutations. After the RD-chip analysis discarded all known RD genes, Whole Exome Sequencing (WES) was undertaken in two pedigrees. The pathogenic mutations were unexpectedly identified in two RD candidates, which had been previously discarded as non-cosegregating on the basis of homozygosity by descent in consanguineous families, and infrequent recombination of closely mapping SNPs.

Methods

Patients

Thirty-six Spanish families diagnosed with RP, LCA, CRD or CD plus 5 isolated Usher's syndrome patients were recruited for this study. Written informed consent from the patients and relatives was obtained following the tenets of the Declaration of Helsinki. Patient recruitment and sample collection procedures had been previously approved by the Bioethics Committee of the University of Barcelona (Barcelona, Spain). Peripheral blood DNA was obtained using the MoleStrips DNA Blood kit with the GeneMole instrument (Mole Genetics, Mole, Lysaker, Norway). DNA from Spanish control individuals was obtained from peripheral blood using the same methodology.

SNP Selection

The RD chip for the molecular diagnosis of Mendelian non-syndromic retinal dystrophies was an optimized version of a previous cosegregation chip for RP-LCA disorders [12,13]. Seven to ten SNPs were selected for each candidate (100 genes in total), and genotyped on a customized Golden Gate Genotyping Assay (Illumina). The SNPs were selected following: i) high informativity according to SNPbrowser Software Version 4.0.1 and dbSNP database (www.nlm.nih.gov/projects/SNP/); ii) physical location (covering upstream, intragenic and downstream regions); iii) inclusion in different haplotypic blocks. The genes analyzed by this RD chip are listed by chromosome position in Figure 1. In addition, some common mutations in *ABCA4, CERKL, COL8A2, CRB1, LRP5, NR2E3, PRPF31, RHO* and *USH2A*, were included for direct genotyping.

High-throughput SNP Genotyping

One microgram of sample DNA (at 20 ng/µl) was laid in 96-well plates. SNPs were genotyped using the Golden Gate Array (Illumina) platform following the instructions, protocol and software provided by the manufacturers. Haplotype and cosegregation analyzes were performed by hand from the raw data genotypes.

Mutational Screening

In Usher syndrome samples, where a major causative gene is involved, the Arrayed Primer Extension (APEX) approach plus direct gene sequencing was the molecular diagnosis procedure of selection. In all other cases, cosegregation analysis with the RD-chip allowed to highlight the best candidates for mutational screening. All the exons and exon–intron boundaries of selected genes were directly screened for mutations in each patient. Genomic DNA was amplified, purified on High Pure 96 UF Cleaning Plates (Roche) and sequenced using the BigDye v3.1 kit (Applied Biosystems, Inc.) in the ABI PRISM 3730 DNA sequencer (Applied Biosystems, Inc.).

All missense changes identified were verified in control population using the dbSNP database (Build 137, www.ncbi.nlm.nih.gov/projects/SNP/), the 1000 Genomes Project data (http://browser.1000genomes.org/index.html), and ESP6500 data of the National Heart, Lung, and Blood Institute GO Exome Sequencing Project (http://evs.gs.washington.edu/EVS). To validate unreported missense genetic variants, over one hundred matched controls were analyzed to discard rare non-pathogenic polymorphisms restricted to the Spanish population.

Bioinformatic Analyses

All the sequences were analyzed using the sequence assembly software Seqman (DNAStar, Madison, WI) and aligned to the reference gene sequence (Genome Reference Consortium human genome build 37, human genome 19).

The pathogenicity of all new missense changes identified in patients was evaluated using the *in silico* predictors SIFT (http://sift.jcvi.org/[15]) and PolyPhen-2 (http://genetics.bwh.harvard.edu/pph2/[16]). When the putative mutations affected splice sites, the splicing site score values of the wild-type and variant sequences were predicted online with NetGene2 (http://www.cbs.dtu.dk/services/NetGene2/[17,18]), Human Splicing Finder [19], and MaxEntScan (http://www.umd.be/HSF/[20]) prediction servers.

Whole-Exome Sequencing

Genomic DNA from peripheral blood was prepared using the QIAamp DNA Blood Maxi Kit (Qiagen). Exome capture was performed at the CNAG using SureSelectXT All Exome v4 kit (Agilent), following the manufacturer's protocol. Libraries were sequenced on a HiSeq2000 (Illumina) to at least 80x average coverage of the target region.

Reads were aligned to the human reference genome build GRCh37 (hg19) using the Burrows-Wheeler Aligner (BWA) [21] in family E5 and GEMMapper [22] in family 9RE. Mapped reads were filtered (leaving only those mapping in unique genomic positions with enough quality), sorted and indexed with SAMtools (version 0.1.18) [23]. Identification of single nucleotide variants and Indels was performed using GATK standard hard filtering parameters [24] (family E5) or SnpEff [25] (family 9RE). In house Perl scripts were used to select the variants shared by all affected individuals, predicted to produce a high or moderate impact, including intron-exon junctions, non-annotated variants (synonymous, non-synonymous, and non-sense mutations) in coding regions, or short coding insertions or deletions. Variants mapping to the candidate genes were selected for further validation. For the final WES report the VARIANT [26] annotation tool provided the putative functional consequence, as well as other additional relevant information of the identified variants for the final candidate gene selection.

Chr	Gene	Covered region (hg18)	CD,CRD	CSNB	LCA	MD	RP	OR
1	RPE65	68.415.679 - 68.909.560	X		X		X	
1	ABCA4	94.222.719 - 94.431.982	X			X	X	
1	GNAT2	109.942.500 - 109.972.419						X
1	PRPF3	148.532.328 - 148.602.797					X	
1	SEMA4A	154.387.714 - 154.467.069	X				X	
1	HMCN1	183.966.670 - 184.434.556				X		
1	CFH	194.842.225 - 195.059.087				X		
1	CRB1	195.301.449 - 195.833.465	X		X		X	X
1	RD3	209.633.383 - 209.770.830			X			
1	USH2A	214.242.873 - 214.830.563					X	
2	ZNF513	27.438.170 - 27.466.535					X	
2	C2ORF71	29.134.249 - 29.152.859					X	
2	EFEMP1	55.941.781 - 56.012.150				X		
2	FAM161A	61.891.702 - 61.954.848					X	
2	SNRNP200	96.288.996 - 96.343.840					X	
2	CNNM4	96.787.353 - 96.904.965						X
2	CNGA3	98.333.140 - 98.430.085						X
2	MERTK	112.365.943 - 112.712.281	X		X		X	
2	CERKL	181.860.076 - 182.241.456	X				X	
2	SAG	233.846.587 - 234.011.399		X			X	
3	GNAT1	50.198.597 - 50.223.811		X				
3	IMPG2	102.398.652 - 102.537.708					X	
3	RHO	130.533.633 - 131.033.267		X			X	
3	OPA1	194.782.019 - 194.905.089						X
4	PDE6B	424.079 - 805.055		X			X	
4	PROM1	15.577.672 - 15.692.948	X			X	X	
4	CNGA1	47.374.685 - 47.884.898					X	
4	LRAT	155.873.871 - 155.924.594			X		X	
4	CYP4V2	187.346.860 -187.385.127						X
5	VCAN	82.798.333 - 82.914.835						X
5	PDE6A	149.218.109 - 149.427.821					X	
5	GRM6	178.328.456 - 178.362.981		X				
6	TULP1	35.530.732 - 35.816.612			X		X	
6	GUCA1A	42.206.073 - 42.267.535	X				X	
6	GUCA1B	42.256.658 - 42.305.894				X	X	
6	PRPH2	42.752.601 - 42.861.382	X			X	X	
6	EYS	66.096.322 - 66.352.493					X	
6	RIMS1	72.646.723 - 73.168.175	X					
6	LCA5	80.233.216 - 80.336.597			X			
6	ELOVL4	80.667.558 - 80.731.013				X		
7	KLHL7	23.106.624 - 23.186.975					X	
7	RP9	33.082.770 - 33.139.684					X	
7	TSPAN12	120.213.054 - 120.299.196						X
7	IMPDH1	127.760.369 - 127.876.209			X		X	
7	OPN1SW	128.195.549 - 128.208.371						X
8	RP1L1	10.496.410 - 10.552.688				X		
8	ADAM9	38.923.050 - 39.165.544	X					
8	RP1	55.674.229 - 55.756.140					X	
8	CNGB3	87.627.399 - 87.838.015	X			X		X
9	KCNV2	2.700.537 - 2.725.415	X					
9	TOPORS	32.516.233 - 32.563.602					X	
10	RBP3	47.988.279 - 48.043.081					X	
10	CDHR1	85.908.918 - 85.968.919	X				X	
10	RGR	85.950.129 - 86.124.939					X	X
10	RBP4	95.328.584 - 95.370.959						X
10	PDE6C	95.358.506 - 95.419.747	X					
10	OAT	126.064.223 - 126.142.968						X
11	TEAD1	12.738.235 - 12.929.811						X
11	BEST1	61.447.283 - 61.502.867				X		X
11	ROM1	62.123.562 - 62.216.570					X	
11	CABP4	66.962.038 - 66.991.927	X	X				
11	LRP5	67.830.957 - 68.014.437						X
11	TMEM126A	84.988.923 - 85.072.109						X
11	FZD4	86.322.657 - 86.357.222						X
11	C1QTNF5	118.690.557 - 118.722.665				X		
11	MFRP	118.708.715 - 118.723.922						X
12	CACNA2D4	1.769.975 - 1.904.551	X					
12	RDH5	54.375.476 - 54.469.113	X	X				
12	CEP290	86.934.473 - 87.125.195			X			
13	RB1	47.737.742 - 47.957.578						X
13	GRK1	113.350.294 - 113.485.357		X				
14	RPGRIP1	20.661.565 - 21.063.338	X		X			
14	NRL	23.455.999 - 23.661.732			X		X	
14	OTX2	56.319.531 - 56.367.417			X			
14	RDH12	67.136.312 - 67.431.984			X		X	
14	SPATA7	87.896.594 - 88.016.375			X		X	
14	TTC8	88.356.598 - 88.415.263					X	
15	TRPM1	29.077.739 - 29.185.374		X				
15	NR2E3	69.524.477 - 70.353.669	X				X	X
15	RLBP1	87.482.020 - 87.674.801					X	X
16	CNGB1	56.311.769 - 56.576.754					X	
16	CDH3	67.227.572 - 67.303.342						X
17	PRPF8	1.457.008 - 1.568.927					X	
17	AIPL1	6.252.280 - 6.303.111	X		X			
17	PITPNM3	6.295.689 - 6.402.825	X					
17	GUCY2D	7.580.474 - 7.956.738	X		X		X	
17	UNC119	23.885.279 - 23.923.629	X					
17	CA4	55.371.053 - 55.937.320					X	
17	RGS9	60.578.935 - 60.674.048						X
17	PRCD	72.030.753 - 72.132.418					X	
17	FSCN2	77.057.292 - 77.185.156				X	X	
17	PDE6G	77.207.216 - 77.291.775					X	
19	RAX2	3.706.266 - 3.734.070	X			X		
19	RGS9BP	37.839.497 - 37.891.623						X
19	CRX	52.942.132 - 53.261.521	X		X		X	
19	PRPF31	59.245.509 - 59.338.102					X	
20	IDH3B	2.569.998 - 2.604.328					X	
22	TIMP3	31.521.373 - 31.589.104				X		
X	RPGR	37.992.315 - 38.101.193	X			X	X	
X	RP2	46.549.608 - 46.671.740					X	

Figure 1. Genes included in the RD-chip. The RD-chip contains the most relevant RD genes at the time of the array design (100 genes). Genes are listed by chromosome and physical location. The interval between the first and last genotyped SNP is shown as ''Covered region'' (the chromosome position is based on human reference sequence NCBI 36/hg18). Colored boxes indicate association with particular retinal dystrophies. CD/CRD: Cone or Cone-Rod Dystrophy; CSNB: Congenital Stationary Night Blindness; LCA: Leber Congenital Amaurosis; MD: Macular Degeneration; RP: Retinitis Pigmentosa; OR: Other Retinopathies.

doi:10.1371/journal.pone.0088410.g001

RT-PCR Expression Analysis

Blood samples from patients, relatives and unrelated controls were mixed with an RNA stabilizer solution (RNALater; Ambion) in a 1:4 ratio. Total RNA was obtained from 3 ml of blood using the RiboPure-Blood Kit (Ambion, Austin, TX), and retrotranscribed using the Transcriptor High Fidelity cDNA Synthesis Kit (Roche Applied Science, Indianapolis, IN) with a mixture of random hexamers and oligo(dT)18, according to the manufacturer's instructions. *RPGRIP1* and *G3PDH* (used as control) transcripts were amplified using specific exon primers and the GoTaq Flexi DNA polymerase (Promega, Fitchburg, WI) in a final volume of 50 µl. The *G3PDH* PCR conditions were: denaturation for 5 min at 94°C followed by 35 cycles of 20 s at 94°C, 30 s at 60°C, and 1 min at 72°C, using 2 µl of cDNA. For *RPGRIP1*, primers were located in exons 14 and 16, and the PCR conditions were: denaturation for 5 min at 94°C followed by 38 cycles of 20 s at 94°C, 30 s at 60°C and 40 s at 72°C, using 5 µl of cDNA. Amplified bands were excised, purified from the gel using the Expin GeneAll Gel SV kit (GeneAll) following the manufacturer's protocol, and sequenced.

Plasmid Constructions and Expression Assay

The reconstructed *RPGRIP1* minigene encompassed exons 12 to 18, plus at least 200 bp of each intron-exon boundary, after amplification of genomic DNA from patient 1 of the 59RE pedigree (heterozygote for the c.2367+23del mutation). The minigene was cloned in-frame at the C-terminus of the HA epitope into the pcDNA3.1 expression vector. Wild-type and mutated clones were confirmed by sequencing.

HEK293 cells were seeded on 12-well plates (4×10^5 cells/well) and grown in DMEM (Invitrogen, Barcelona, Spain) supplemented with 10% of fetal bovine serum. After 12 hours, cells were transiently transfected with, either the pcDNA3.1-wild type (wt) *RPGRIP1* minigene, the pcDNA3.1-mut *RPGRIP1* minigene (containing the c.2367+23del mutation), or the empty vector (Clontech-BD), using Lipofectamine 2000 (Invitrogen). Forty-eight hours after transfection, cells were collected, lysed, and total mRNA was used for RT-PCR (same protocol as above). *RPGRIP1* cDNA amplification was performed using primers of exons 15 and 18 as follows: denaturation for 5 min at 94°C, followed by 35 cycles of 20 s at 94°C, 30 s at 62°C, and 1 min 30 s at 72°C, using 1 µl of cDNA. *G3PDH* amplification was used as a control.

Results

Classification of the Patients According to Phenotype and Family Information

Our initial cohort comprised 41 families, 36 affected with non-sydromic RDs and 5 affected by Usher syndrome type II. We attained full diagnosis for 22 of them, whose pedigrees are displayed in Figure 2. Cosegregation with the novel mutated alleles identified is shown in Figure S1.

Analysis of Usher Syndrome Cases

Usher syndrome is characterized by specific phenotypic traits that allow a clear clinical characterization in three main forms, being USH II the most frequent type (between half and two thirds of all cases), and *USH2A* the major causative gene (75–80% of USH II cases) [27]. *O*ur cohort had 5 USH II cases as isolated patients or small pedigrees, which when directly tested for described mutations in *USH2A* (Asper Biotech), only 4 out of 10 mutated alleles were identified, all in heterozygosis. As no complete molecular diagnosis was achieved, direct mutational screening of all *USH2A* exons was then undertaken, prioritizing the analysis of exons where reported mutations cluster. Overall, three of the five patients (pedigrees shown in Figure 2) were completely diagnosed, with 3 missense mutations identified: c.1751G>T in heterozygosis (p.C584F), c.10636G>A in homozygosis (p.G3546R), and c.12574C>T in heterozygosis (p.R4192C) (Table 1 A). The pathogenicity of the two novel variants, p.C584F and p.R4192C, was supported by the PolyPhen and SIFT algorythms (Table 2). In the remaining two patients only one mutated allele was identified (Table 1 A).

Genotyping of RD Families

Contrary to Usher syndrome, most RDs show high clinical and genetic heterogeneity, which greatly hampers molecular diagnosis. Our approach was to use genetic information and cosegregation analysis to decrease the number of candidate genes for mutational screening. When pedigrees were available, the use of an automated and robust SNP-based genotyping microarray greatly diminished the number of candidates. To this aim, 36 families (with at least four available samples) affected with retinal dystrophies (Retinitis Pigmentosa, Leber Congenital Amaurosis, Cone-rod Dystrophy or Cone Dystrophy) were analyzed with our optimized in house RD-chip that genotyped 768 SNP markers spanning the 100 most prevalent RD genes reported at that moment (Figure 1). Six families showed autosomal dominant inheritance and a large number of affected individuals; twenty-nine were autosomal recessive pedigrees with a low number of affected members, and the remaining two were compatible with X-linked inheritance.

After RD-chip genotyping, haplotypes were constructed for each family to assess cosegregation under the presumed inheritance pattern. In pedigrees where 90 to 99% of candidates were discarded, direct mutational screening was performed in the remaining non-excluded genes. For each case, the candidates were prioritized according to previous gene assignment to: 1) the same clinical diagnosis and mendelian pattern; 2) a closely related retinal dystrophy with the same inheritance pattern; 3) the same clinical phenotype irrespective of the inheritance pattern, and finally 4) the remaining RD candidates.

This approach allowed us to identify the pathogenic mutation in 17 families out of 36 (47,2%), depicted in Figure 2:2/2 in X-linked families, 6/18 of recessive non-consanguineous families (33,3%), 8/10 recessive consanguineous families (80%) and 1/6 of dominant families (16,6%).

Inferred Haplotypes and Subsequent Mutation Screening of the Prioritized Candidates in X-linked and Dominant Pedigrees

The genotyping results for each family were first analyzed under the most probable mendelian pattern of inheritance to exclude non-cosegregating genes and prioritize the remaining candidates. The final results are presented by family and summarized in Table 1 B.

Seven pedigrees were compatible with an autosomal dominant (ad) pattern, but one (11NCE) could also be explained by an X-linked inheritance, as all affected women showed a less severe phenotype. In this case, the haplotypes were first analyzed under a X-linked hypothesis (see below). Of the six AD families, only 2 (6ORG and 2NCE) were amenable for mutational screening, while in the rest more than 10 candidates remained. Indeed, in dominant diseases a large number of samples is required to attain genetic informativity, which seldom occurs. In family 6ORG, the RD-chip highlighted seven candidates, three of them responsible for adRP: *SEMA4A*, *SNRNP200* and *TOPORS*. We prioritized the

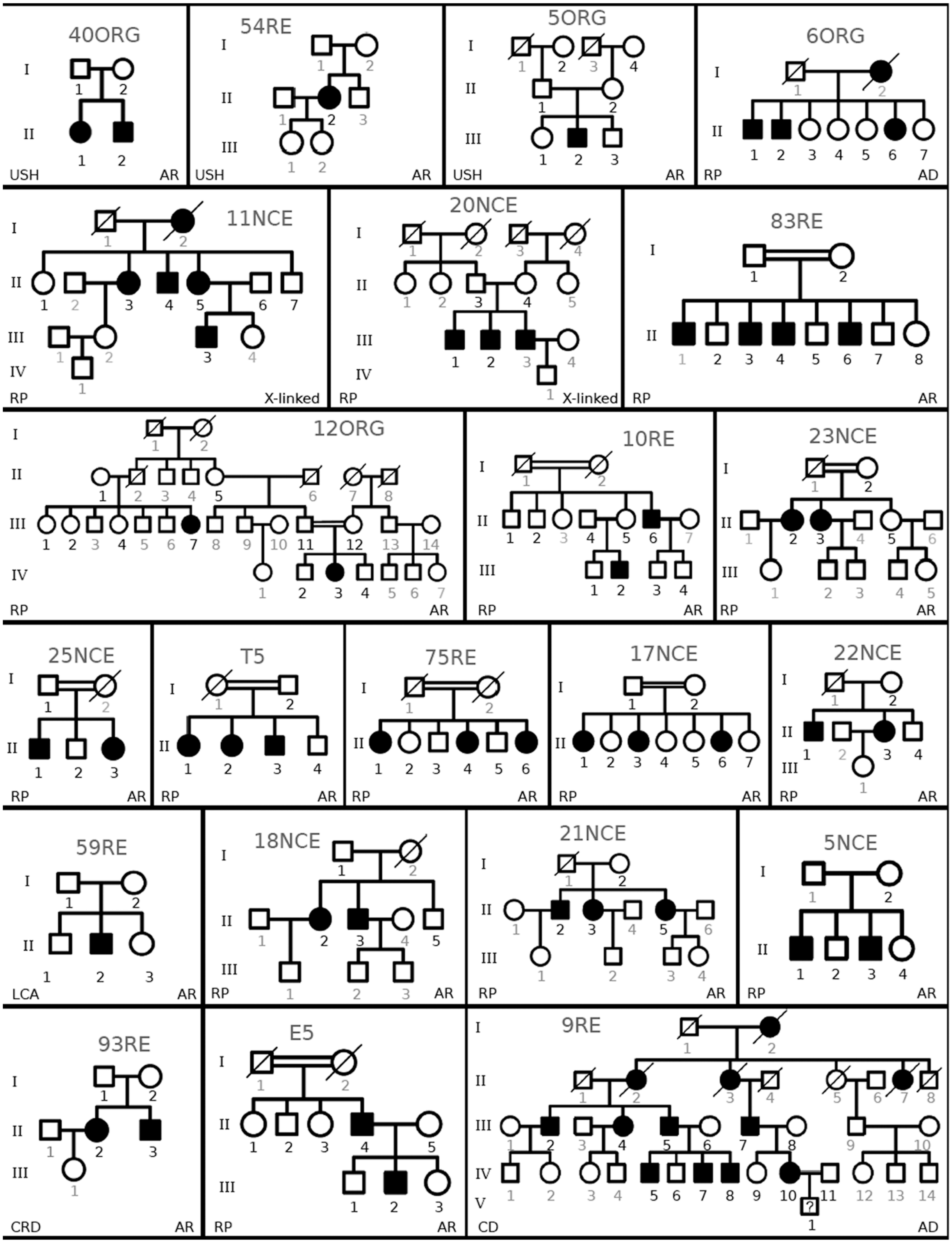

Figure 2. Spanish pedigrees diagnosed in this study. Families were categorized as arUSH II, arRP, adRP, XL-RP, arLCA, arCRD and adCD according to mendelian pattern of inheritance and phenotype. Bold numbers indicate samples available for analysis.
doi:10.1371/journal.pone.0088410.g002

Table 1. Summary of the diagnosed families in this study.

Family	Phenotype	Inheritance	Gene	Nucleotide change	Protein change	Zygosity	References
**A) USH II families**							
40ORG	Usher II	recessive	*USH2A*	c.1751G>T	p.C584F	Het	[a]
				c.2299delG[b]	p.E767Sfs*20	Het	[50]
54RE	Usher II	recessive	*USH2A*	c.10636G>A	p.G3546R	Hom	[34]
5ORG	Usher II	recessive	*USH2A*	c.9799T>C[b]	p.C3267R	Het	[33]
				c.12574C>T	p.R4192C	Het	[a]
56RE	Usher II	recessive	*USH2A*	c.2299delG[b]	p.E767Sfs*20	Het	[50]
				n.i	n.i		
94RE	Usher II	recessive	*USH2A*	c.2299delG[b]	p.E767Sfs*20	Het	[50]
				n.i	n.i		
**B) Families analized by the RD-xip with identified mutations (sorted by gene).**							
12ORG	RP	recessive	*CRB1*	c.1702C>T	p.H568Y	Hom	[a]
10RE	LCA	recessive	*CRB1*	c.3749+2_3749+3del	*splicing*	Homo/Het	[a]
				c.2843G>A	p.C948Y	Het	[30]
23NCE	RP	recessive	*CRB1*	c.2290C>T	p.R764C	Hom	[30]
25NCE	RP	recessive	*CRB1*	c.2843G>A	p.C948Y	Hom	[30]
T5	RP	recessive	*CRB1*	c.2843G>A	p.C948Y	Hom	[30]
17NCE	RP	recessive	*MERTK*	c.2189+1G>T	*splicing*	Hom	[31]
22NCE	RP	recessive	*PROM1*	c.1984-1G>T	*splicing*	Hom	[a]
83RE	RP	recessive	*RD3*	c.259A>G	p.K87E	Hom	[a]
11NCE	RP	X-linked	*RP2*	c.409_411del	p.I137del	Hemi	[29]
20NCE	RP	X-linked	*RP2*	All gene deletion		Hemi	[a]
59RE	LCA	recessive	*RPGRIP1*	c.895_896del	p.E299Sfs*21	Het	[a]
				c.2367+23del[c]	intronic	Het	[a]
6ORG	RP	dominant	*SNRNP200*	c.2042G>T	p.R681L	Het	[a]
18NCE	RP	recessive	*USH2A*	c.2276G>T	p.C759F	Het	[32]
				c.9799T>C	p.C3267R	Het	[33]
21NCE	RP	recessive	*USH2A*	c.1434G>C	p.E478D	Het	[51]
				c.2276G>T	p.C759F	Het	[32]
75RE	RP	recessive	*USH2A*	c.2209C>T	p.R737X	Het	[52]
				c.8693A>C	p.Y2898S	Het	[a]
5NCE	RP	recessive	*USH2A*	c.652-2A>G	*splicing*	Het	[a]
				c.2276G>T	p.C759F	Het	[32]
93RE	CRD	recessive	*ABCA4*	c.3988G>T	p.E1330X	Het	[a]
				c.6410G>A	p.C2137Y	Het	[a]
**C) Families with identified mutations by WES**							
9RE	CD	dominant	*GUCY2D*	c.2747T>C	p.I916T	Het	[a]
E5	RP	recessive	*USH2A*	c.2167+5G>A	*splicing*	Het	[53]
				c.4325T>C	p.F1442S	Het	[35]
				c.7364G>A	p.W2455X	Het	[a]

For each family, the phenotype, inheritance model, the altered gene, the identified mutation, the homozygosity/heterozygosity state, and the reference of previously described mutations are indicated. [a] This study, [b]Mutations previously identified by APEX [c]Unknown pathogenicity, [n.i] Not identified.
doi:10.1371/journal.pone.0088410.t001

analysis of *SNRNP200*, as the cosegregating haplotype extended to three neighbouring RD genes (*CNNM4, CNGA3, MERTK*), overall covering more than 16 Mb (Figure 1). Direct exonic sequence revealed a novel missense mutation in *SNRNP200*, c.2042G>T (p.R681L) (Table 1 B) in a codon also mutated in other adRP cases (c.2041C>T, p.R681C and c.2042G>A, p.R681H) [28]. Bioinformatics analysis showed that this residue was highly evolutionary conserved (data not shown) and predicted a damaging effect (Table 2). Moreover, none of the healthy siblings did carry the pathogenic variant, supporting its pathogenicity. In family 2NCE, after the RD chip analysis, ten candidates remained. Extended haplotypes with neighbouring RD genes decreased the number of candidates to five. Unfortunately, no mutation was found in any candidate (the pedigree is not included in Figure 2).

Table 2. Pathogenicity predictions for new missense mutations.

		SIFT		PolyPhen-2	
**Gene**	**Mutation**	**Score**	**Prediction**	**Score**	**Prediction**
CRB1	p.H568Y	1	Tolerated	0,999	Probably damaging
GUCY2D	p.I916T	0,002	Damaging	1	Probably damaging
RD3	p.K87E	0,01	Damaging	0,997	Probably damaging
SNRNP200	p.R681L	0	Damaging	1	Probably damaging
USH2A	p.C584F	0	Damaging	1	Probably damaging
USH2A	p.F1442S	0	Damaging	1	Probably damaging
USH2A	p.Y2898S	0	Damaging	0,998	Probably damaging
USH2A	p.R4192C	0	Damaging	0,998	Probably damaging
ABCA4	p.C2137Y	0	Damaging	1	Probably damaging

Dash(−) means no splice site predicted.
doi:10.1371/journal.pone.0088410.t002

X-linked inheritance was assumed for 20NCE and 11NCE, the latter being also compatible with AD inheritance. SNP genotyping revealed a common deleted region comprising the full *RP2* locus in all male patients of 20NCE, clearly underscoring *RP2* as the disease-causing gene. Indeed, mutational screening confirmed the deletion of the whole coding region. Concerning pedigree 11NCE, the milder affectation of women -suggestive of an X-linked trait-, added to the cosegregation of the *RP2* haplotype, both pinpointed this candidate for mutational screening. Exon sequencing identified a previously reported mutation, c.409_411del causing p.I137del, in all the family patients (Table 1 B) [29].

Recessive Consanguineous Families

In five of ten known consanguineous families (10RE, 12ORG, 23NCE, T5, and 25NCE), *CRB1* was the candidate of choice. Prioritization was established based on either an extended haplotype comprising the adjacent *CFH* locus in four pedigrees, or a shared haplotype with a previously diagnosed family, suggesting a founder effect.

The patient II.6 in the family 10RE (Figure 2) carried an homozygous unreported deletion in the intron 9 splice donor site of *CRB1*, c.3749+2_3749+3del, which ablated the splice signal. This outcome was confirmed by *in silico* predictions (Table 3). His nephew was a double heterozygote for this mutation plus a frequent pathogenic variant, c.2843G>A p.C948Y [30].

In family 12ORG, all but four RD genes were discarded, of those, *CRB1* showed an extended haplotype. Direct sequencing revealed a novel missense mutation in homozygosity, c.1702C>T p.H568Y, whose pathogenicity was supported by *in silico* analyses (Table 2). On the other hand, families 23NCE and T5 showed homozygosity for the known missense mutations c.2290C>T p.R764C, and c.2843G>A p.C948Y, respectively [30]. Of note, the haplotype of family T5 affected members was also shared by the patients of another family (25NCE). Subsequent sequencing analysis of *CRB1* confirmed the same causative mutation, supporting common ancestry.

Family 83RE showed an extended haplotype spanning *USH2A* and *RD3*. Given that the clinical diagnosis of the family was RP, *USH2A* was prioritized for direct mutational screening but was excluded after sequencing 72 exons. Screening of the *RD3* candidate, previously reported only as a LCA-causative gene, revealed a new missense mutation c.259A>G p.K87E in homozygosity. *In silico* predictions supported its pathogenicity (Table 2). Notably, this variant had been identified in 4 out of 13.002 control chromosomes (NHLBI Exome Sequencing Project).

The consanguineous family 75RE was first analyzed assuming homozygosity by descent, and under this assumption, all candidates were discarded. However, if non-consanguinity was assumed an extended haplotype spanning candidates *USH2A* and *RD3* emerged. Subsequent mutational screening of *USH2A* identified two disease-causing mutations in the patients: the reported nonsense c.2209C<T p.R737X and the novel missense c.8693A>C p.Y2898S. The pathogenicity of the latter was fully supported by *in silico* predictions (Table 2).

The non-discarded genes of family 17NCE were prioritized according to: 1) cosegregation and phenotype, pointing to *LRAT*, or 2) shared haplotype with a previously diagnosed Spanish family in *MERTK* [31]. *LRAT* was discarded after sequencing of all exons, whereas direct screening of *MERTK* exon 16 identified the expected mutation c.2189+1G>T in homozygosis, again supporting a founder effect.

Two remaining families were not further considered due to the lack of genetic informativity.

Recessive Non-consanguineous Families

After cosegregation analysis, three candidate genes remained in the 22NCE family (*PROM1*, *RP1* and *TEAD1)*. Although consanguinity had not been reported, the mutational screening of *PROM1* revealed a novel homozygous mutation, c.1984-1G>T, which ablates the consensus acceptor splice site of intron 17. Its pathogenicity was fully confirmed by *in silico* predictions (Table 3).

In family 59RE, seven candidate genes cosegregated, although the clinical phenotype of the patients pointed *RPGRIP1* and *TULP1* as the best candidates. *TULP1* did not bear any mutation, whereas two previously unreported variants in *RPGRIP1* were identified. The variant c.895_896del, p.E299Sfs*21, was clearly pathogenic and produced a truncated protein. The other variant was intronic, c.2367+23del, and the possible pathogenic effect was unknown. *In silico* predictions for splice sites, splice enhancers and silencers did not reveal any strong molecular alteration. Besides, *in vivo* analysis of patient's mRNAs was restrained by the *RPGRIP1* low expression levels in blood. Finally, the transfection in cultured cells of minigenes bearing either the WT or the variant sequence did not conclusively support its pathogenicity. As the analysis of 434 control chromosomes identified this variant once, the c.2367+23del variant could be presumably classified as a rare indel, and its pathogenicity remains to be proved.

Table 3. Pathogenicity predictions for new splicing mutations.

		NetGene2 (0–1)	MaxEnt (score)	HSF (0–100)
CRB1	wt	0,37	9,6	96,67
	c.3749+2_3749+3del	–	–	–
MERTK	wt	0,86	5,58	86,8
	c.2189+1G>T	–	–	–
PROM1	wt	0	8,36	84,3
	c.1984−1G>T	–	–	–
USH2A	wt	0,83	4,89	90,92
	c.652−2A>G	–	0,56	–

doi:10.1371/journal.pone.0088410.t003

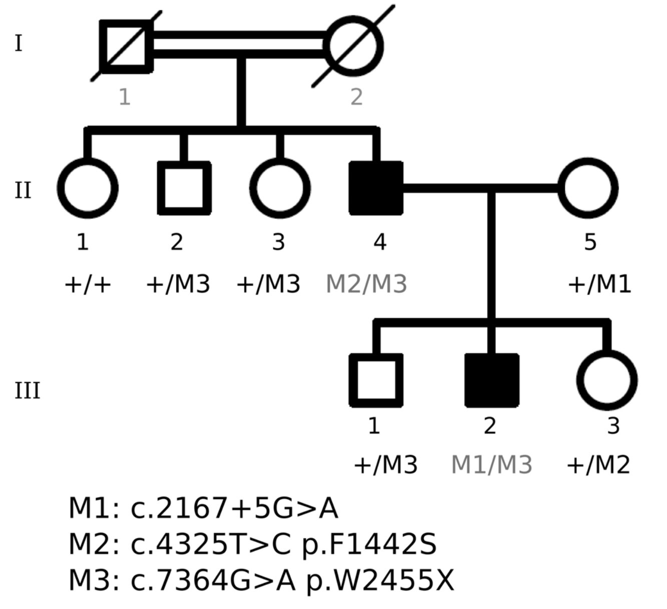

Figure 3. Cosegregation of *USH2A* mutations identified in family E5. Three heterozygous *USH2A* mutations cosegregate with the disease in this consanguineous family. Patient II-4 carried two mutated alleles M2 (c.4325T>C) and M3 (c.7364G>A), whereas his affected son (III-2) inherited the M3 paternal allele plus the reported M1 (c.2167+5G>A) mutation from his mother.
doi:10.1371/journal.pone.0088410.g003

Three families compatible with a recessive RP inheritance (5NCE, 18NCE and 21NCE) showed cosegregation with 5–12 candidates, *USH2A* among them. Given that direct genotype of mutations in the RD-chip had already detected a frequent *USH2A* pathogenic allele c.2276G>T, p.C759F, in heterozygosis [32], direct sequencing of the full coding sequence was undertaken. Data revealed one novel pathogenic allele in family 5NCE, c.652-2A>C, which ablates the acceptor splice site, and two reported mutations c.9799T>C p.C3267R (18NCE) and c.1434G>C p.E478D (21NCE); although the pathogenicity of this last variant is still controversial [32–34].

Family 93RE whose clinical diagnosis was compatible with either CRD or recessive Stargardt disease showed cosegregation with *GNAT2*, *ZNF513*, *OPA1*, *RP1L1* and *ABCA4*. Based on the type of inheritance and phenotype, we prioritized the analysis of *GNAT2*, *ZNF513* and *ABCA4*. Two unreported mutations in *ABCA4* were identified, the nonsense c.3988G>T p.E1330X and the missense c.6410G>A p.C2137Y variants. *In silico* predictions by PolyPhen2 and SIFT of the latter supported its pathogenicity (Table 2).

WES Families

After exclusion with the chip of the one hundred RD candidates, WES was undertaken in suitable remaining families. Concerning family E5, most recessive RD genes were discarded under the assumption of claimed consanguinity (I.1 and I.2), and the rest of candidates was excluded by Sanger sequencing. WES was then undertaken for patients II.4 and III.2 (Figure 2). Unexpectedly, one novel (c.7364G>A) and one recently reported mutation (c.4325T>C) [35] in *USH2A* were identified in patient II.4, whereas his affected son (patient III.2) carried the c.4325T>C mutation from his father plus the reported pathogenic c.2167+5G>A allele inherited from his mother. Haplotype analysis of the pedigree confirmed cosegregation of these pathogenic variants (Figure 3). In this family, the exceptional non-compliance with the homozygosity-by-descent assumption had excluded *USH2A* as the causative gene in the RD chip analysis.

The 9RE pedigree (Figure 2) showed an autosomal dominant cone dystrophy. In this large cohort, the high genetic informativity allowed us to exclude most candidates after the RD cosegregation chip, and Sanger sequencing ruled out the few remaining genes. Exome sequencing was then undertaken and identified a new mutation in *GUCY2D*, c.2747T>C. This candidate had not been previously considered because the SNP haplotype analysis showed recombination within the 3′ flanking region in one affected member (IV 5). Although the selected SNPs are closely linked to the genes to minimize it, recombination, however infrequent, may occur and mislead the analysis.

RD-Chip Efficiency

Table 4 summarizes the percentage of complete molecular diagnosis attained with the RD chip, categorized by inheritance pattern and the number of samples available per family. Six autosomal dominant families with at least 4 available samples (and a minimum of three patients) were analyzed, yet only one family, 6ORG, (7 samples with three affected members) was successfully diagnosed. Indeed, when dealing with dominant diseases, the main drawback for successful diagnosis is the large number of samples required to attain discriminative genetic informativity. In contrast, in recessive families, the percentage of success doubles to 33% (6 out of 18) in non-consanguineous, and dramatically raises up to 80% (8 out of 10), in consanguineous pedigrees. We conclude that the highest informativity of recessive pedigrees, particularly under the homozygosity-by-descent assumption in consanguinity, increases the efficiency of the RD chip and highlights its reliability for RD molecular diagnosis.

Table 4. Families solved by the RD-chip according to the number of samples available and the inheritance pattern.

	Number of families solved/Total number of families						
	**4 samples**	**5 samples**	**6 samples**	**7 samples**	**8 samples**	**>8 samples**	**Total**
Dominants		0/1	0/1	1/2		0/2	**1/6**
Recessives	4/7*	2/6	0/1	0/2	0/1	0/1	**6/18**
Consanguineous	2/2	1/3	1/1		2/2	2/2	**8/10**
X-linked	1/1			1/1			**2/2**
**Total**	7/10	3/10	1/3	2/5	2/3	2/5	**17/36**

*One allel identified by direct genotiping in 3 cases.
doi:10.1371/journal.pone.0099354.t004

Table 5. Comparison of efficiencies of different methods for RD molecular diagnosis.

	N° Genes analyzed	Yield	Reference
APEX	1-16	15-44%	[44, 54]
Long-PCR	9	33%	[43]
Autozygome	16-100	42-52%	[48, 55]
Target Capture	105-179	36-56%	[11, 44-47, 56]
WES	All	44-83%*	[43, 48, 49, 57]
WGS	All	50%	[10]
RD-chip	100	47%	This study

*Higher efficiency percentages are obtained when few families or cases are analysed.
doi:10.1371/journal.pone.0099354.t005

Finally, two X-linked cohorts were successfully diagnosed. The RD chip included markers for cosegregation analysis of the two known X-linked causative loci (*RP2* and *RPGR*), given that X-linked inheritance can only be discarded in pedigrees with male-to-male transmission [36], and as a means to avoid the burdensome task of sequencing the hot-spot ORF15 of *RPGR* whenever cosegregation analysis does not support it.

Discussion

To meet the increasing demand for genetic diagnosis from clinicians and patients, we have devised and optimized an RD chip that allows us to extract maximum genetic informativity from pedigrees, exclude a large number of non-cosegregating candidates and focus on the most probable causative genes. Moreover, the exclusion of all analyzed candidates by the RD chip highlights the families suitable for next generation sequencing (NGS) and subsequent identification of novel RD genes.

To improve the efficiency and the scope of the RD-Chip with respect previous attempts [12,37] we have: i) increased the number of SNPs (7–10) per gene to maximize the genetic informativity, ii) extended the cosegregation study to 100 retinal dystrophy genes, and iii) included some prevalent pathogenic point mutations in the Spanish population for direct genotyping. The RD-chip allows to genotype 768 selected SNPs in a single cost- and time-effective step and is designed to use on families, not on simplex cases.

With this optimized RD-chip version, 17 out of 36 Spanish pedigrees have been fully diagnosed. Twelve new and 12 previously reported pathological variants have been identified in 9 RD genes, adding to the high genetic diversity in retinal disorders. The fact that as much as half of the mutations identified are new underscores the efficiency of our RD chip compared to direct mutational screening., particularly in non-homogenous genetic populations. Concerning the major candidates in our cohort, *CRB1* and *USH2A* explain 10/19 families overall, in accordance with other reports [38,39] and consistent with their contribution to several clinical entities. *CRB1* is responsible for RP and LCA (a more severe form of RP), and more than 150 mutations have been described so far (http://www.hgmd.org). In our panel, the LCA pedigree (10RE) carried a novel splicing mutation, whereas the RP pedigrees (12ORG, 23NCE, 25NCE, T5) were all associated to missense variants (Table 1). Our results agree with the *CRB1*-assigned phenotype-genotype correlations, where null alleles are mainly associated to the LCA phenotype [39,40]. Also in agreement with previous reports, p.C948Y is the most prevalent *CRB1* mutation in our family panel (3 of the 5 *CRB1* families) [39].

Five non-syndromic RP families presented 4 new (2 missense, one nonsense and one splicing mutation) and 7 reported (5 missense, one nonsense and one splicing mutation) pathogenic alleles in *USH2A*. In the Usher cohorts, analysis of *USH2A* rendered 2 new missense and 3 known (one frameshift and 2 missense) mutations. In two families, only one of the pathogenic alleles was identified. In fact, some reports support that as much as 35% of the second *USH2A* mutant alleles are duplications, deletions and deep intronic variants, which are extremely difficult to detect by DNA sequencing [27]. On the other hand, no clear phenotype-genotype correlation could be established between RP and Usher cases. The most prevalent *USH2A* mutation in non-syndromic RP, p.C759F [32,41], was also present in three families of our cohort. Notably, double heterozygosis in *USH2A* was unexpectedly found in two consanguineous families. In fact, in pedigree E5, three pathogenic alleles were identified in two generations. In this particular case, the assumption of homozygosity by descent led us to wrongly assume non-cosegregation with all RD genes and undertake WES analysis, which eventually identified an unexpected double heterozygous genotype. A seemingly higher number of mutation carriers had also been reported for other syndromic RPs, such as Bardet-Biedl, with no solid evidence for this finding [42].

The inclusion of X-linked markers in the RD-chip has proved to be extremely useful to diagnose families compatible with both autosomal and X-linked inheritance patterns. In fact, in pedigree 11NCE, the milder affectation of the female patients was already suggestive of a pseudo-dominant effect, as it was indeed confirmed (*RP2* was the causative gene). On the other hand, family 20NCE, with an unassigned mendelian pattern, cosegregated with X-linked markers, which prompted to focus on the X-linked candidates. A deletion comprising the *RP2* locus was identified (Table 1).

Remarkably, the clinical heterogeneity of retinal disorders was highlighted by the identification of *RD3*–up to now associated only to LCA– as causative of RP (Table 1), increasing the phenotypes associated to the gene mutations. This case would have remained unassigned by conventional methods had it been not for the comprehensive analysis of our RD chip, whose main asset is the robustness of genetic information to highlight the most probable candidates, avoiding the yet burdensome task of big data analysis. Particularly, identification of shared (which indicate a common founder effect) or extended haplotypes over closely mapping genes (which strengthen cosegregation) are invaluable clues to directly pinpoint the causative mutation, unveil unexpected candidates, and/or prompt re-evaluation of clinical features.

Recently, a variety of NGS-based procedures have been developed for molecular diagnosis of RDs, from targeted long-

range PCR coupled to NGS [43,44], targeted capture and sequencing of one or several RD genes [45–47], to full WES analysis [11,48,49]. Although powerful tools, they still yield limited complete diagnostic success: from 37%–52% in non-related cohorts to 80% in cohorts with high consanguinity levels [48] (Table 5). Aside high costs, the restraints of NGS-targeted approaches are due to the high genetic heterogeneity of retinal disorders, whereas the main drawbacks of WES are the high sequence coverage requirement and the functional interpretation of the identified variants (WES). Within this context, the RD-chip efficiency ranges from 16% in dominant to 80% in consanguineous recessive pedigrees, with an average of 47%, well within the upper range of the NGS approaches. Besides, the design of this chip is extremely flexible, which allows to incorporate new SNPs to expand the gene repertoire after new discoveries or upon demand. Before NGS paves the future of personalized diagnosis, our cost- and time-effective strategy allows a quick and reliable prioritization of candidates, which is suitable and affordable for middle-size diagnostic labs with moderate to high number of family cases.

Supporting Information

Figure S1 Cosegregation analysis of the novel mutations identified. M: mutation
(TIF)

Acknowledgments

We gratefully acknowledge all patients and their relatives for participating in this study. We are obliged to A. Mayor for sample collection, helpful discussions and constant support to our research. We are indebted to B. Cormand for his guidance and invaluable advice on WES. We also thank P. Méndez and S. Ruiz for technical assistance.

Author Contributions

Conceived and designed the experiments: MdCM EP GM RGD. Performed the experiments: MdCM EP LLM. Analyzed the data: MdCM EP LLM RT JD GM RGD. Contributed reagents/materials/analysis tools: RT JD GM RGD. Wrote the paper: MdCM GM RGD.

References

1. Retinal Information Network website Available: https://sph.uth.edu/RetNet/.
2. Athanasiou D, Aguila M, Bevilacqua D, Novoselov SS, Parfitt DA, et al. (2013) The cell stress machinery and retinal degeneration. FEBS Lett 587: 2008–2017.
3. Andrade LJ, Andrade R, Franca CS, Bittencourt AV (2009) Pigmentary retinopathy due to Bardet-Biedl syndrome: case report and literature review. Arq Bras Oftalmol 72: 694–696.
4. Bonnet CEl-Amraoui A (2012) Usher syndrome (sensorineural deafness and retinitis pigmentosa): pathogenesis, molecular diagnosis and therapeutic approaches. Curr Opin Neurol 25: 42–49.
5. Estrada-Cuzcano A, Roepman R, Cremers FP, den Hollander AI, Mans DA (2012) Non-syndromic retinal ciliopathies: translating gene discovery into therapy. Hum Mol Genet 21: R111–124.
6. Stenson PD, Ball EV, Mort M, Phillips AD, Shaw K, et al. (2012) The Human Gene Mutation Database (HGMD) and its exploitation in the fields of personalized genomics and molecular evolution. Curr Protoc Bioinformatics Chapter 1: Unit1 13.
7. den Hollander AI, Black A, Bennett J, Cremers FP (2010) Lighting a candle in the dark: advances in genetics and gene therapy of recessive retinal dystrophies. J Clin Invest 120: 3042–3053.
8. Berger W, Kloeckener-Gruissem B, Neidhardt J (2010) The molecular basis of human retinal and vitreoretinal diseases. Prog Retin Eye Res 29: 335–375.
9. Koenekoop RK, Lopez I, den Hollander AI, Allikmets R, Cremers FP (2007) Genetic testing for retinal dystrophies and dysfunctions: benefits, dilemmas and solutions. Clin Experiment Ophthalmol 35: 473–485.
10. Nishiguchi KM, Tearle RG, Liu YP, Oh EC, Miyake N, et al. (2013) Whole genome sequencing in patients with retinitis pigmentosa reveals pathogenic DNA structural changes and NEK2 as a new disease gene. Proc Natl Acad Sci U S A 110: 16139–16144.
11. Neveling K, Collin RW, Gilissen C, van Huet RA, Visser L, et al. (2012) Next-generation genetic testing for retinitis pigmentosa. Hum Mutat 33: 963–972.
12. Pomares E, Marfany G, Brion MJ, Carracedo A, Gonzalez-Duarte R (2007) Novel high-throughput SNP genotyping cosegregation analysis for genetic diagnosis of autosomal recessive retinitis pigmentosa and Leber congenital amaurosis. Hum Mutat 28: 511–516.
13. Pomares E, Riera M, Permanyer J, Mendez P, Castro-Navarro J, et al. (2010) Comprehensive SNP-chip for retinitis pigmentosa-Leber congenital amaurosis diagnosis: new mutations and detection of mutational founder effects. Eur J Hum Genet 18: 118–124.
14. Pomares E, Marfany G, Gonzalez-Duarte R (2012) High-throughput approaches for the genetic diagnosis of retinal dystrophies. Adv Exp Med Biol 723: 329–335.
15. Ng PC, Henikoff S (2001) Predicting deleterious amino acid substitutions. Genome Res 11: 863–874.
16. Adzhubei IA, Schmidt S, Peshkin L, Ramensky VE, Gerasimova A, et al. (2010) A method and server for predicting damaging missense mutations. Nat Methods 7: 248–249.
17. Brunak S, Engelbrecht J, Knudsen S (1991) Prediction of human mRNA donor and acceptor sites from the DNA sequence. J Mol Biol 220: 49–65.
18. Hebsgaard SM, Korning PG, Tolstrup N, Engelbrecht J, Rouze P, et al. (1996) Splice site prediction in Arabidopsis thaliana pre-mRNA by combining local and global sequence information. Nucleic Acids Res 24: 3439–3452.
19. Desmet FO, Hamroun D, Lalande M, Collod-Beroud G, Claustres M, et al. (2009) Human Splicing Finder: an online bioinformatics tool to predict splicing signals. Nucleic Acids Res 37: e67.
20. Yeo G, Burge CB (2004) Maximum entropy modeling of short sequence motifs with applications to RNA splicing signals. J Comput Biol 11: 377–394.
21. Li H, Durbin R (2009) Fast and accurate short read alignment with Burrows-Wheeler transform. Bioinformatics 25: 1754–1760.
22. Marco-Sola S, Sammeth M, Guigo R, Ribeca P (2012) The GEM mapper: fast, accurate and versatile alignment by filtration. Nat Methods 9: 1185–1188.
23. Li H, Handsaker B, Wysoker A, Fennell T, Ruan J, et al. (2009) The Sequence Alignment/Map format and SAMtools. Bioinformatics 25: 2078–2079.
24. DePristo MA, Banks E, Poplin R, Garimella KV, Maguire JR, et al. (2011) A framework for variation discovery and genotyping using next-generation DNA sequencing data. Nat Genet 43: 491–498.
25. Cingolani P, Platts A, Wang le L, Coon M, Nguyen T, et al. (2012) A program for annotating and predicting the effects of single nucleotide polymorphisms, SnpEff: SNPs in the genome of Drosophila melanogaster strain w1118; iso-2; iso-3. Fly (Austin) 6: 80–92.
26. Medina I, De Maria A, Bleda M, Salavert F, Alonso R, et al. (2012) VARIANT: Command Line, Web service and Web interface for fast and accurate functional characterization of variants found by Next-Generation Sequencing. Nucleic Acids Res 40: W54–58.
27. Steele-Stallard HB, Le Quesne Stabej P, Lenassi E, Luxon LM, Claustres M, et al. (2013) Screening for duplications, deletions and a common intronic mutation detects 35% of second mutations in patients with USH2A monoallelic mutations on Sanger sequencing. Orphanet J Rare Dis 8: 122.
28. Benaglio P, McGee TL, Capelli LP, Harper S, Berson EL, et al. (2011) Next generation sequencing of pooled samples reveals new SNRNP200 mutations associated with retinitis pigmentosa. Hum Mutat 32: E2246–2258.
29. Sharon D, Bruns GA, McGee TL, Sandberg MA, Berson EL, et al. (2000) X-linked retinitis pigmentosa: mutation spectrum of the RPGR and RP2 genes and correlation with visual function. Invest Ophthalmol Vis Sci 41: 2712–2721.
30. den Hollander AI, ten Brink JB, de Kok YJ, van Soest S, van den Born LI, et al. (1999) Mutations in a human homologue of Drosophila crumbs cause retinitis pigmentosa (RP12). Nat Genet 23: 217–221.
31. Brea-Fernandez AJ, Pomares E, Brion MJ, Marfany G, Blanco MJ, et al. (2008) Novel splice donor site mutation in MERTK gene associated with retinitis pigmentosa. Br J Ophthalmol 92: 1419–1423.
32. Rivolta C, Sweklo EA, Berson EL, Dryja TP (2000) Missense mutation in the USH2A gene: association with recessive retinitis pigmentosa without hearing loss. Am J Hum Genet 66: 1975–1978.
33. Aller E, Jaijo T, Beneyto M, Najera C, Oltra S, et al. (2006) Identification of 14 novel mutations in the long isoform of USH2A in Spanish patients with Usher syndrome type II. J Med Genet 43: e55.
34. Garcia-Garcia G, Aparisi MJ, Jaijo T, Rodrigo R, Leon AM, et al. (2011) Mutational screening of the USH2A gene in Spanish USH patients reveals 23 novel pathogenic mutations. Orphanet J Rare Dis 6: 65.
35. Mendez-Vidal C, Gonzalez-Del Pozo M, Vela-Boza A, Santoyo-Lopez J, Lopez-Domingo FJ, et al. (2013) Whole-exome sequencing identifies novel compound heterozygous mutations in USH2A in Spanish patients with autosomal recessive retinitis pigmentosa. Mol Vis 19: 2187–2195.
36. Churchill JD, Bowne SJ, Sullivan LS, Lewis RA, Wheaton DK, et al. (2013) Mutations in the X-linked retinitis pigmentosa genes RPGR and RP2 found in 8.5% of families with a provisional diagnosis of autosomal dominant retinitis pigmentosa. Invest Ophthalmol Vis Sci 54: 1411–1416.
37. Permanyer J, Navarro R, Friedman J, Pomares E, Castro-Navarro J, et al. (2010) Autosomal recessive retinitis pigmentosa with early macular affectation caused by premature truncation in PROM1. Invest Ophthalmol Vis Sci 51: 2656–2663.
38. Hartong DT, Berson EL, Dryja TP (2006) Retinitis pigmentosa. Lancet 368: 1795–1809.

39. Bujakowska K, Audo I, Mohand-Said S, Lancelot ME, Antonio A, et al. (2011) CRB1 mutations in inherited retinal dystrophies. Hum Mutat 33: 306–315.
40. den Hollander AI, Davis J, van der Velde-Visser SD, Zonneveld MN, Pierrottet CO, et al. (2004) CRB1 mutation spectrum in inherited retinal dystrophies. Hum Mutat 24: 355–369.
41. Aller E, Najera C, Millan JM, Oltra JS, Perez-Garrigues H, et al. (2004) Genetic analysis of 2299delG and C759F mutations (USH2A) in patients with visual and/or auditory impairments. Eur J Hum Genet 12: 407–410.
42. Hjortshoj TD, Gronskov K, Philp AR, Nishimura DY, Riise R, et al. (2010) Bardet-Biedl syndrome in Denmark–report of 13 novel sequence variations in six genes. Hum Mutat 31: 429–436.
43. Licastro D, Mutarelli M, Peluso I, Neveling K, Wieskamp N, et al. (2012) Molecular diagnosis of Usher syndrome: application of two different next generation sequencing-based procedures. PLoS One 7: e43799.
44. Fujinami K, Zernant J, Chana RK, Wright GA, Tsunoda K, et al. (2013) ABCA4 Gene Screening by Next-Generation Sequencing in a British Cohort. Invest Ophthalmol Vis Sci 54: 6662–6674.
45. Chen X, Zhao K, Sheng X, Li Y, Gao X, et al. (2013) Targeted sequencing of 179 genes associated with hereditary retinal dystrophies and 10 candidate genes identifies novel and known mutations in patients with various retinal diseases. Invest Ophthalmol Vis Sci 54: 2186–2197.
46. Huang XF, Xiang P, Chen J, Xing DJ, Huang N, et al. (2013) Targeted exome sequencing identified novel USH2A mutations in Usher syndrome families. PLoS One 8: e63832.
47. Glockle N, Kohl S, Mohr J, Scheurenbrand T, Sprecher A, et al. (2013) Panel-based next generation sequencing as a reliable and efficient technique to detect mutations in unselected patients with retinal dystrophies. Eur J Hum Genet:.
48. Abu-Safieh L, Alrashed M, Anazi S, Alkuraya H, Khan AO, et al. (2013) Autozygome-guided exome sequencing in retinal dystrophy patients reveals pathogenetic mutations and novel candidate disease genes. Genome Res 23: 236–247.
49. Corton M, Nishiguchi KM, Avila-Fernandez A, Nikopoulos K, Riveiro-Alvarez R, et al. (2013) Exome sequencing of index patients with retinal dystrophies as a tool for molecular diagnosis. PLoS One 8: e65574.
50. Eudy JD, Weston MD, Yao S, Hoover DM, Rehm HL, et al. (1998) Mutation of a gene encoding a protein with extracellular matrix motifs in Usher syndrome type IIa. Science 280: 1753–1757.
51. Seyedahmadi BJ, Rivolta C, Keene JA, Berson EL, Dryja TP (2004) Comprehensive screening of the USH2A gene in Usher syndrome type II and non-syndromic recessive retinitis pigmentosa. Exp Eye Res 79: 167–173.
52. Kaiserman N, Obolensky A, Banin E, Sharon D (2007) Novel USH2A mutations in Israeli patients with retinitis pigmentosa and Usher syndrome type 2. Arch Ophthalmol 125: 219–224.
53. Najera C, Beneyto M, Blanca J, Aller E, Fontcuberta A, et al. (2002) Mutations in myosin VIIA (MYO7A) and usherin (USH2A) in Spanish patients with Usher syndrome types I and II, respectively. Hum Mutat 20: 76–77.
54. Avila-Fernandez A, Cantalapiedra D, Aller E, Vallespin E, Aguirre-Lamban J, et al. (2010) Mutation analysis of 272 Spanish families affected by autosomal recessive retinitis pigmentosa using a genotyping microarray. Mol Vis 16: 2550–2558.
55. Alazami AM, Alshammari MJ, Salih MA, Alzahrani F, Hijazi H, et al. (2012) Molecular characterization of Joubert syndrome in Saudi Arabia. Hum Mutat 33: 1423–1428.
56. Wang X, Wang H, Sun V, Tuan HF, Keser V, et al. (2013) Comprehensive molecular diagnosis of 179 Leber congenital amaurosis and juvenile retinitis pigmentosa patients by targeted next generation sequencing. J Med Genet 50: 674–688.
57. Neveling K, Feenstra I, Gilissen C, Hoefsloot LH, Kamsteeg EJ, et al. (2013) A Post-Hoc Comparison of the Utility of Sanger Sequencing and Exome Sequencing for the Diagnosis of Heterogeneous Diseases. Hum Mutat 34: 1721–1726.
